# Using Baidu Search Index to Predict Dengue Outbreak in China

**DOI:** 10.1038/srep38040

**Published:** 2016-12-01

**Authors:** Kangkang Liu, Tao Wang, Zhicong Yang, Xiaodong Huang, Gabriel J Milinovich, Yi Lu, Qinlong Jing, Yao Xia, Zhengyang Zhao, Yang Yang, Shilu Tong, Wenbiao Hu, Jiahai Lu

**Affiliations:** 1 School of Public Health, Key Laboratory of Tropical Diseases Control of Ministry of Education, Sun Yat-Sen University, Guangzhou, P. R. China; 2 Zhongshan Research Institute of Public Health, School of Public Health, Sun Yat-Sen University, Zhongshan, P. R. China; 3 School of Public Health and Social Work, Institute of Health and Biomedical Innovation, Queensland University of Technology, Brisbane, Australia; 4 Zhongshan Center for Diseases Control and Prevention, Zhongshan, P. R. China; 5 Guangzhou Center for Disease Control and Prevention, Guangzhou, P. R. China; 6 School of Medicine, University of Queensland, Brisbane, Australia; 7 School of Public Health, University at Albany, State University of New York, Albany, United States; 8 The School of Food Science, Guangdong Pharmaceutical University, Guangzhou, P. R. China; 9 Institute of Emergency Technology for Serious Infectious Diseases Control and Prevention, Guangdong Provincial Department of Science and Technology; Emergency Management Office, the People’s Government of Guangdong Province, Guangzhou, P. R. China

## Abstract

This study identified the possible threshold to predict dengue fever (DF) outbreaks using Baidu Search Index (BSI). Time-series classification and regression tree models based on BSI were used to develop a predictive model for DF outbreak in Guangzhou and Zhongshan, China. In the regression tree models, the mean autochthonous DF incidence rate increased approximately 30-fold in Guangzhou when the weekly BSI for DF at the lagged moving average of 1–3 weeks was more than 382. When the weekly BSI for DF at the lagged moving average of 1–5 weeks was more than 91.8, there was approximately 9-fold increase of the mean autochthonous DF incidence rate in Zhongshan. In the classification tree models, the results showed that when the weekly BSI for DF at the lagged moving average of 1–3 weeks was more than 99.3, there was 89.28% chance of DF outbreak in Guangzhou, while, in Zhongshan, when the weekly BSI for DF at the lagged moving average of 1–5 weeks was more than 68.1, the chance of DF outbreak rose up to 100%. The study indicated that less cost internet-based surveillance systems can be the valuable complement to traditional DF surveillance in China.

Dengue fever (DF) is a major public health concern, particularly in the tropical and sub-tropical regions. The incidence of DF has increased 30-fold over the last five decades; between 1990 and 2013, dengue has been estimated to account for approximately ten thousand deaths per year^1^,^2^. As a fast spreading vector-borne infectious disease, DF is endemic to over 120 countries^3^. Recent estimates indicate that 390 million people suffer DF each year, including 96 million new cases^4^; this estimate is three times that of the DF burden estimated by WHO in 2009^5^. In China, DF cases have been reported in Guangdong, Hainan, Fujian, Yunnan, and Zhejiang every year, since the first DF outbreak in 1978. The burden of DF has increased with a number of large outbreaks occurring over the previous ten years; the most recent major outbreak resulted in over 40,000 cases in Guangdong province in 2014.

Prevention and control of DF primarily focuses on case surveillance, vector control, and DF vaccine initiatives. Although the first DF vaccine was registered in 2015 in Mexico^3^, however, further testing of the vaccines efficacy need to be performed to allow its use in other countries. Traditional surveillance systems for DF are built on the basis of passive or sentinel site surveillance in the outpatient services or hospitals. These systems are limited by underreporting, delayed diagnosis and under-resourced laboratory services which limits case confirmation^6^. The development of real-time and accurate infectious disease surveillance remains a real challenge worldwide.

Digital surveillance systems that are built on internet search engines data can provide health authorities with important information regarding the emergence and spread of diseases in the community which can be used to complement traditional healthcare-based surveillance systems^7^. For example, Google Flu Trend (http://www.google.org/flutrends/) was used in the accurate real-time tracking of influenza outbreaks in some studies^8^,^9^,^10^. Moreover, the real-time detection and prediction using the internet-based surveillance systems have also been explored in some other diseases such as Ebola, malaria, and breast cancer^11^,^12^,^13^. One novel method for exploring early disease detection is based on monitoring of health-seeking behavior using the internet search engines. Although some studies^12^,^14^ have presented the benefits of internet search query data on diseases for improving real-time tracking and surveillance systems, this innovative approach requires further development.

Google is not available in China. The majority of web searches originating from China are submitted through the Baidu search engine (http://www.baidu.com/). It was reported by China Internet Network Information Center (CNNIC) that the total number of internet users using Baidu search engine was up to 649 million, accounting for 47.9% of the national population in 2014^15^. Baidu is the most popular search engine in China and the Baidu search volume is provided as a weighted index, available to the public called Baidu Search Index (BSI) (http://index.baidu.com). The occurrences of some infectious diseases, including influenza, gonorrhea, and erythromelalgia have been demonstrated to be associated with BSI for certain search terms^16^,^17^,^18^. However, DF activity in China has not been explored and tracked with the web search behavior.

This study aimed to identify the association between BSI and DF, and detect the possible threshold of DF outbreaks with machine-learning method time-series classification and regression trees (CART) model based on Baidu search query data. CART models are decision algorithms that incorporate traditional regression models. These models have been extensively used in the public health field^19^,^20^ as they provide a robust approach to explore complex, non-linear ecological data. We propose that CART models could provide the threshold analysis for the early detection and prediction of diseases such as DF to facilitate clinical decision making^20^,^21^.

## Results

### Descriptive Analysis

The summary statistics are depicted in Table 1 for the autochthonous DF cases and Baidu search query data between 1^st^ January 2010 and 31^st^ December 2014 in Guangzhou and Zhongshan. During the study period, 38,866 autochthonous DF cases and 771 imported cases were reported for Guangzhou; 1,476 autochthonous cases and 167 imported DF cases were reported in Zhongshan city. The weekly mean autochthonous DF weekly cases were 148.91 (range: 0 to 8,201) in Guangzhou and 5.68 (range: 0 to 154) in Zhongshan during the study period. The peak weekly counts of autochthonous DF cases were 8,201 during the period of 29^th^ September 2014 to 5^th^ October 2014 in Guangzhou and 154 during the period of 6^th^ October 2014 to 12^th^ October 2014 in Zhongshan.

**Figure 1:**
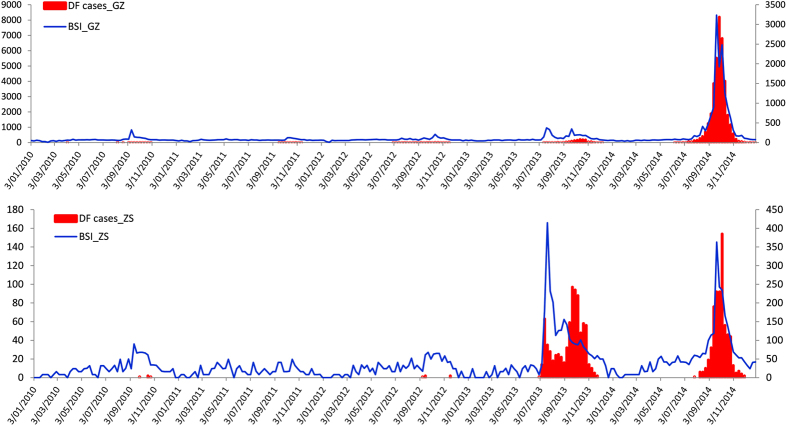
Weekly distribution of DF cases and Baidu search query data in Guangzhou, 2010–2014.

The weekly trends of autochthonous DF cases and Baidu search query data are shown from 1^st^ January 2010 and 31^st^ December 2014 in Guangzhou and Zhongshan (Fig. 1). There were similar trends between the weekly counts of autochthonous DF cases and the weekly BSI for DF in Guangzhou and Zhongshan during the study period, while the peak of BSI for DF was somewhat different from the peak of autochthonous DF cases during the period between July 2013 and October 2013 in Zhongshan. The largest outbreaks occurred in 2014 in both Guangzhou and Zhongshan.

**Figure 2:**
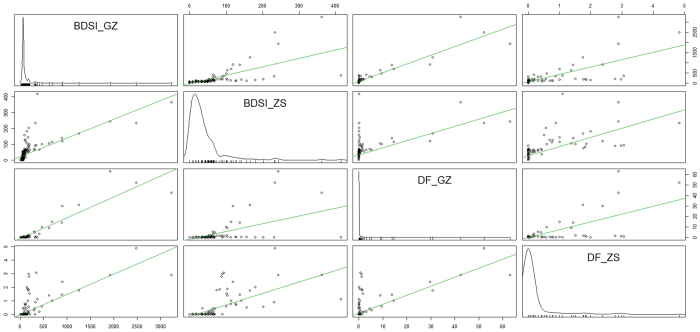
Scatterplot matrix among DF incidence rates and Baidu search query data in Guangzhou and Zhongshan, 2010–2014.

The results of Spearman’s correlation reveal weekly DF incidence rates to be positively associated with the weekly BSI for DF in Guangzhou (*ρ* = *0.687, P* < *0.01*) and Zhongshan (*ρ* = *0.800, P* < *0.01*) (Table S3). The scatterplot matrix with the regression lines in Fig. 2 displays the relationships between the weekly DF incidence rates and the weekly BSI for DF in Guangzhou and Zhongshan.

### Cross-correlations analysis

**Figure 3:**
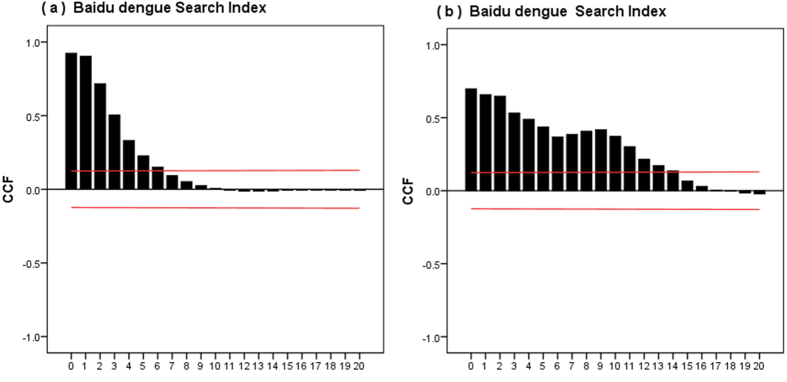
Cross-correlation between DF incidence rates and Baidu search query data. (**a**) The cross-correlation between DF incidence rate and Baidu search data in Guangzhou from 2010 to 2014; (**b**) The cross-correlation between DF incidence rate and Baidu search data in Zhongshan from 2010 to 2014. CCF: Cross-Correlation Function; The two dashed lines illustrate critical values for cross-correlation (at the 5% level).

Cross-correlation analysis indicates that the weekly autochthonous DF incidence rates are positively correlated with the weekly BSI for DF at the time lags of 1–6 weeks in Guangzhou (Fig. 3). There is also a positive correlation between weekly DF incidence rates and weekly BSI for DF at the lags of 1–14 weeks in Zhongshan. Cross-correlation coefficients at a range of lags of the variables BSI for DF which were more than 0.4 were chosen in two cities, respectively. BSI for DF at the lags of 1–3 weeks in Guangzhou and BSI for DF at the lags of 1–5 weeks in Zhongshan, were selected as the independent variable of CART models.

### CART analysis

#### Regression Tree

**Figure 4:**
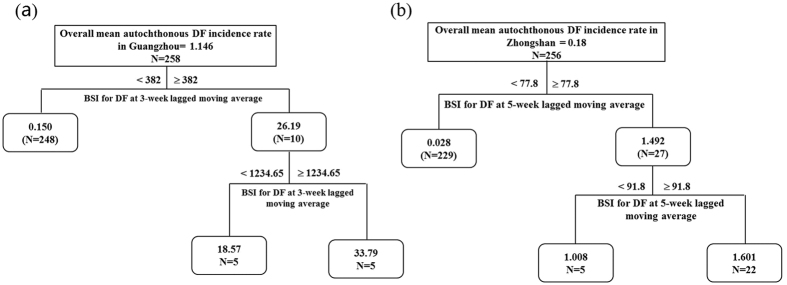
The regression tree modeling the hierarchical relationship between weekly autochthonous DF incidence and Baidu search query data in Guangzhou and Zhongshan, China between 1 January 2010 and 31 December 2016. (**a**) The regression tree in Guangzhou; (**b**) The regression tree in Zhongshan. The regression tree showed the threshold values, mean weekly autochthonous DF incidence rate, N is the total week count of occurrence of DF outbreaks.

Figure 4 reveals that the mean autochthonous DF incidence rate increased by approximately 30-fold in Guangzhou (mean autochthonous DF incidence rate 34.45 compared to an overall mean weekly autochthonous DF incidence rate 1.146) when the weekly BSI for DF at moving average of 1–3 weeks ≥382. Moreover, when the weekly BSI for DF at the lagged moving average of 1–3 weeks was ≥382 and <1234.65, the weekly autochthonous DF incidence rate decreased by approximately 16-fold (to a mean autochthonous DF incidence rate 18.57 relative to the overall mean autochthonous DF incidence rate 1.146). The mean autochthonous DF incidence rate was only 0.15 if the BSI for DF was <382 at the lagged moving average of 1–3 weeks week. Figure 4 indicates that, for Zhongshan, if the weekly BSI for DF at the lagged moving average of 1–5 weeks was <91.8 and ≥77.8, the mean autochthonous DF incidence rate increased by 5.6-fold. When the BSI for DF at the lagged moving average of 1–5 weeks was ≥91.8, there was approximately 9-fold increase of the mean autochthonous DF incidence rate. The mean weekly incidence rate of the autochthonous DF was only 0.028 if the 5-week lagged moving average of the weekly BSI for DF was <77.8, compared to the overall mean weekly autochthonous DF incidence rate. The results of regression tree model indicate the threshold value of the occurrence of DF outbreak can be considered for use as a DF epidemic early warning tool and decision-making by the health department of the government.

#### Classification tree

**Figure 5:**
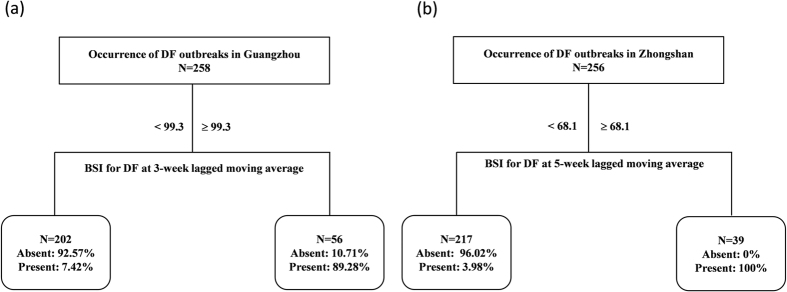
The classification tree modeling the ordered relationship between DF weekly incidence rate and Baidu Search Index in Guangzhou and Zhongshan, China from 1 January 2010 to 31 December 2014. (**a**) The classification tree in Guangzhou; (**b**) The classification tree in Zhongshan. The classification tree showed the threshold values, mean weekly autochthonous DF incidence rate, N is the total week count of occurrence of DF.

The results of classification tree in Fig. 5 indicate that the probability of the occurrence of DF epidemic in Guangzhou and Zhongshan. It reveals that the optimal classification tree had two terminal nodes for Guangzhou and Zhongshan. For Guangzhou, there was 89.28% chance of the occurrence of DF outbreaks when the weekly BSI for DF at the lagged moving average of 1–3 weeks was ≥99.3. The probability of the occurrence of DF outbreaks was only 7.42% when the weekly BSI for DF at the lagged moving average of 1–3 weeks was <99.3. For Zhongshan, when the lagged moving average at 1–5 weeks of the weekly BSI for DF was ≥68.1, the chance of DF outbreak rose up to 100%; while, if the lagged moving average at 1–5 weeks of the weekly BSI for DF was <68.1, there was only 3.98% probability for the DF outbreak (Fig. 5). The accuracy of the classification tree models was evaluated in Table 2. The consistency rate of the classification tree model is 91.57 in Guangzhou and that is 94.636 in Zhongshan, China.

## Discussion

Internet-based surveillance systems have been increasingly explored, in recent years, as an innovative approach to improving the effectiveness of infectious diseases prevention and control programs. For example, the online digital diseases surveillance tool based on Google Trends and Google Insight have been mined and reported by some studies^8^,^22^,^23^. Little work has, however, focused on the use of Baidu. This study is the first attempt to investigate the possibility and application of the Baidu search query data in the timely and sensitive detection of DF activities in China. Our results have clearly shown a positive significant relationship between the occurrence of DF outbreaks and Baidu search query data and provided a possibility for the further prediction of DF occurrence in advance using search query surveillance data. When the weekly historical BSI for DF at the lagged moving average of 1–5 weeks was more than 382, the incidence rate of autochthonous DF may increase 30-fold compared with the overall mean autochthonous DF incidence rate in Guangzhou. When the BSI for DF at the lagged moving average of 1–5 weeks was more than 91.8, there was approximately 9-fold increase of the mean autochthonous DF incidence rate in Zhongshan. These results indicate the benefit for the early intervention and control for DF outbreak by the health authorities and decision-maker of public health strategies.

Effective control of DF is contingent on high quality, timely surveillance data. Current systems are hindered by delayed and patchy reporting of DF; there is a clear need for better approaches for the early detection and monitoring of DF epidemics^24^. Google Flu Trend demonstrated promise for monitoring and early detection of influenza; it was even able to produce surveillance data up to two weeks before official cases data were reported^8^. A similar approach was applied to DF by Chan *et al*. and Althouse *et al*. These studies demonstrated that search query data based on Google search engine are capable of tracking and predicting the occurrence of DF outbreaks^25^,^26^. Baidu is the main internet search tool used in mainland China. Data generated by Baidu has been applied to surveillance for influenza and erythromelalgia^16^,^18^. However, to our knowledge, few studies have focused on DF epidemics and Baidu search volume data in China. Our study sheds some insights on this issue. Official DF data are generated by passive, low-sensitivity traditional surveillance system and likely under-report cases in China. The key reasons for the underestimation may result from the infected individuals who may not visit medical establishment for clinical diagnosis at the early stages of illness, the lack of medical conditions and policy limitation^27^. The underestimation of DF epidemic could decrease the sensitivity and the accuracy of the traditional surveillance and increase the risk of DF transmission and the complexity of disease control. Internet-based surveillance systems have been shown to be able to circumvent many of the inherent limitations of traditional surveillance systems^7^. Therefore, the online DF-related search query data from the internet users as the supplement tool can provide the chance for the real-time, sensitive detection and control of DF activity before the diagnosis and reports of DF cases. The low-cost and availability of the internet search data is another advantage of this approach. Traditional surveillance approaches are limited by the lag between the time of clinical diagnosis and laboratory testing (confirmation) and reporting of cases. Internet-based approaches may be integrated into surveillance systems to reduce the effect of this lag on the system.

This study combined the novel and modern data sources with the sentinel surveillance data on DF cases, and the results of CART models show that the significant positive relationships are identified between two time series data in Guangzhou and Zhongshan during 2010–2014. Baidu search query data may be used to identify the occurrence of DF outbreak in advance of existing systems. In the current study, the definition of the binary response variables of the classification tree model was similar to the other studies, in which the DF outbreak was defined using the historical DF epidemics data during the study period^26^,^28^. It has been shown that CART models have the advantage of the powerful technology in analysis of the ecological data compared to generalized linear models such as logistic regression model^29^,^30^. CART can provide the threshold values for the potential risk factors or predicted factors of DF epidemic, which are beneficial for the decision-making and strategies setting of public health issues by official government, while the logistic regression model could not achieve^31^. Additionally, CART model are easy to conduct, interpret, and to base decisions on. It is suitable for dealing with missing or zero data analysis, the unspecified interaction variables which lead to multicollinearity, and no linearity assumption which could not be offered by logistic regression analysis. Hence, in the current study, we didn’t take account of the transmission (such as logarithmic transmission or add the number 1 to the count) of the variables which zero observations existed^32^,^33^.

In the present study, considering the incubation period of DF cases and the models for further prediction in advance stably and accurately, though the CART models with a lag of 1 week seemed a little better than the models with lagged moving average of 1–3 weeks in Guangzhou and Zhongshan, respectively (for example, root mean squared error (RMSE) of regression tree model with a lag of 1 week was 3.22, while RMSE of regression tree model with lagged moving average of 1–3 weeks was 3.72 in Guangzhou; RMSE of regression tree model with a lag of 1 week was 0.37, while RMSE of regression tree model with lagged moving average of 1–3 weeks was 0.38 in Zhongshan), we chose the CART models with the lagged moving average of 1–3 weeks in Guangzhou and Zhongshan, instead of the models with the best pure lag of 1 week in the two cities. This study indicates that risk assessments of the threshold effect for the occurrence of DF outbreaks which were performed by the CART models in the two study sites Guangzhou and Zhongshan are presented discrepant (the threshold value of BSI for DF is 99.3 in Guangzhou and which is 68.1 in Zhongshan). The appearances in the two areas may be due to the differences in the social demographic characteristics, the availability, and popularity of the internet, human migration and meteorological factors. The results demonstrated that the models, in general, built on the Baidu search query data for DF may be considered for estimating the occurrence of DF activity linking with official DF data reported by Guangzhou and Zhongshan ministries of health.

The increasing transmission risk of DF outbreak is of concerned in most tropical and subtropical countries. This study responds to the potential possibility and opportunity for the exploration of internet-based surveillance system by Baidu search query data. The identification and prediction of DF outbreaks using is critical to initiating timely public health interventions for DF prevention and control such as mosquito control, clinical treatment, vaccination or education of the targeted population. Prevention and control efforts based on real-time and low-cost internet search data can help official department of public health to identify the target areas which have the potential DF spread risk and take effective measures.

There are two limitations in this study. First, the major challenge of digital surveillance is the unavailability and limitation of internet access in rural areas. The internet penetration rate reported by China Internet Network Information Center (CNNIC) was 47.9% at the end of 2014 in China. The internet users in rural areas account for 27.5% of the internet users overall throughout the country^15^. The web-search based surveillance is built on the internet access rate and search query volume. The internet penetrance of the internet users by computer or mobile device in the rural areas is low, but searching volume from mobile phones in rural areas is increasing due to its convenience and feasibility. As of the end of 2014, 85.8% of the internet users accessed the internet from mobile devices. Guangdong has the internet penetration rate of 68.5% in 2014 (Fig. S2). The second limitation for the internet-based surveillance is that the media reports could impact on the internet search behavior. Not all internet search queries are submitted by patients infected dengue virus. A portion of queries may be from uninfected people who seek the information for other purposes. When a novel outbreak is reported by media, the internet searching behavior would increase by the large proportion of people who are induced by panic or curiosity, which would result in the peak of searching behavior of the disease and the false alert of the internet-based surveillance^8^.

This study found that the Baidu search engine combining with the traditional diseases surveillance system may be considered for early detection of DF activities in China. However, further studies are needed to combine with internet search query data and other potential factors (weather, social and environmental factors) to develop an early warning system (EWS). It will be essential to establish an effective EWS to assist disease control and prevention programs for DF.

## Methods

### Ethics Statement

Ethics clearance for this research was approved by Sun Yat-sen University and Zhongshan Center for Disease Control and Prevention Ethical Review Committee (Approval No: 2015024). DF data were collected from the Notifiable Infectious Disease Report System (NIDRS). All potentially identifiable has been removed to prevent identification of individuals.

### Study sites

In this study, as 94.3% DF cases were identified in Guangdong province in China in the past decades^34^, and the higher internet penetration rates, Guangzhou and Zhongshan in Guangdong province were selected for the study sites. It is reported the internet penetration rate in Guangdong province 72.4%, followed by Beijing and Shanghai. It was reported that internet penetration rates are lower in Yunnan (37.4%) and Hainan (51.6%), compared with the internet penetration rate in Guangdong (72.4%)^15^. Guangzhou is the capital city with the largest population in the south of China and Zhongshan is next to Guangzhou. Most of the occurrence of DF outbreaks happened in Guangzhou, Zhongshan, Foshan and the adjacent cities in Guangdong province recent years, especially the outbreak occurred during 2013–2014. Hence, in the current study, we focus on the mainly two cities Guangzhou and Zhongshan for estimation and prediction of DF.

Guangzhou (latitude 23.117°N, longitude 113.276°E) and Zhongshan city (latitude: 22.515847°N, longitude: 113.392207°E) are located in the south central of Guangdong province, the region of Pearl River Delta, in China. (Fig. S1). The two cities have the similar subtropical monsoonal climate with hot, humid summers and mild, dry sunny winters. The annual mean temperature is between 21–23 °C.

### Data source

#### Epidemiological data

DF is a legally notifiable infectious disease in China and is diagnosed according to the national diagnosis criteria^35^,^36^. The weekly autochthonous DF case count data between 1^st^ January 2010 and 31^st^ December 2014 were obtained from NIDRS. NIDRS is a passive surveillance system of legal notifiable infectious diseases in China. At the city level, autochthonous DF cases were defined as the confirmed cases with no history of travel to dengue-affected foreign countries or other cities in mainland China in the preceding 14 days of the illness onset.

#### Search query data

The shared platform of Baidu index provides search behavior data for numerous search terms with Baidu search engine by internet users. The data are available at national, province, city level by daily and weekly. Given the internet penetrance in the study area and the stability of the platform of Baidu search data, we collected weekly DF-related search queries from the Baidu’s database (http://index.baidu.com) for Guangzhou and Zhongshan from January 2010 to December 2014, respectively. Baidu Search Index which is calculated based on the sum of the weight of search volume is publicly available by certain search term on a daily basis, at a city, province and national level. In this study, we explored and examined whether Baidu search engine could be a valued source of data for the supplementary implement of the traditional healthcare-based DF surveillance. Therefore, we selected the weekly BSI for search term “dengue fever” (i.e. using the Chinese word “
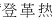
”) which is most widely searched. The weekly DF cases count data are converted from the daily data.

### Statistical analysis and modeling

First, spearman’s correlations analysis (two-tailed test) was used to detect the correlation between the weekly observed autochthonous DF incidence rates and the weekly BSI for DF (key word: “
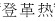
”) in Guangzhou and Zhongshan, respectively. Time-series cross-correlation analysis was used to assess and quantify the linear associations between the two time series data at a function of time lag. In the current study, the two time series dataset including weekly autochthonous DF incidence rates and weekly BSI for DF in Guangzhou and Zhongshan were analyzed over a range of weekly lags, respectively. Given the incubation period of DF, the correlation coefficients of the variables with different lags calculated more than 0.4 with positively significant in results of the cross-correlation analysis were selected in the two cities respectively. Then, in order to explain the lagged effects of the independence variables BSI with the significant lags better, the lagged moving average (MA) of the variables BSI for DF were calculated and transformed to the new variables. The new variables were then put into the model for further analysis. Hence, the correlation coefficients which are at the time lags of 1–3 weeks in Guangzhou and at the time lags of 1–5 weeks are significant and chosen for CART modeling in Guangzhou and Zhongshan, respectively. (Table S1).

CART is a flexible, robust and non-parametric statistical method which is useful for the analysis of complex ecological applications. The CART model is suitable for predicting the relationship between a response variable and one or more exploratory variables^29^. It could interpret the variation of the response variable by recursive partitioning the ecological data into more homogeneous subsets, using combinations of explanatory variables. The response variable can be a categorical variable (a classification tree) or a continuous variable (a regression tree)^37^.

In the study, the regression tree models were built to assess the threshold effects between the weekly DF incidence rates and the weekly BSI for DF in Guangzhou and Zhongshan, respectively. The variation of a response variable was explained using a single explanatory variable which is the lags of 1–3 weeks moving average of BSI for DF in Guangzhou and at the lags of 1–5 weeks moving average of BSI for DF in Zhongshan, respectively in the regression tree model for the purpose of the threshold prediction and explanation.

Meanwhile, the classification tree models were built to determine the threshold effects of the hierarchical relationship between the weekly autochthonous the occurrence of DF outbreak and the weekly BSI for DF in Guangzhou and Zhongshan, respectively. A single explanatory variable in each model was the weekly BSI for DF at the significant at lags of 1–3 weeks moving average of BSI for DF in Guangzhou and at lags of 1–5 weeks moving average of BSI for DF in Zhongshan respectively were chosen to build classification tree models. The binary response variables are the weekly the occurrence of DF outbreaks in the two cities. In order to create the new binary response variables for building classification tree model, we defined the occurrence of DF outbreaks in which weekly DF incidence rates exceeded 75 percentiles in Guangzhou over the study period (Table S2). As the DF incidence rates in Zhongshan is lower than that in Guangzhou, we defined exceeded 85 percentiles of incidence rate for the occurrence of DF outbreak in Zhongshan (Table S2).

The CART was grown by the recursive binary splitting of explanatory variables and then each split form the basis of a single explanatory variable and two nodes, which was selected by the maximize homogeneity (minimize impurity) of the resulting two nodes. The splitting criteria for minimizing node impurity are different in the two types of methods: for regression trees, least squares are used in the process of splitting; for classification trees, the Gini index is used to split off the largest category into a separate subset group. The K-fold cross-validation is performed to cutoff the large tree to the smallest tree size using estimated prediction error rate in this study. The best tree has an estimated error rate within one standard error of the minimum. The process of ten-fold cross-validation in the current study is conducted as following: firstly, all the data were used to build the overly large tree; then, the dataset were divided into ten subsets; nine in ten subsets were used for training, and the subset ten left was used for testing. Hence, the different models were built with the subgroups data with the independent error rates. The best optional tree model was selected by the minimum error rate^32^. The accuracy of the classification tree models including sensitivity, specificity, and consistency rate was calculated and assessed in this study.

All the analyses above were performed using IBM SPSS version 22 (SPSS Inc; Chicago, IL, USA) and R version 3.2.2.

## Additional Information

**How to cite this article**: Liu, K. *et al*. Using Baidu Search Index to Predict Dengue Outbreak in China. *Sci. Rep.* **6**, 38040; doi: 10.1038/srep38040 (2016).

**Publisher's note:** Springer Nature remains neutral with regard to jurisdictional claims in published maps and institutional affiliations.

## Supplementary Material

Supplementary Information

## Figures and Tables

**Table 1:** The summary statistics for DF cases and Baidu search query data in Guangzhou and Zhongshan, China, 2010–2014.

Variables	Mean	SD	Median	Minimum	Maximum
ADF_GZ	148.91	832.74	0.00	0	8201
ADF_ZS	5.68	19.12	0.00	0	154
BSI_GZ	67.00	296.54	67.00	8	3237
BSI_ZS	24.00	50.16	24.00	0	415

ADF_GZ: autochthonous DF cases in Guangzhou; ADF_ZS: autochthonous DF cases in Zhongshan; BSI_GZ: Baidu Search Index for DF in Guangzhou; BSI_ZS: Baidu Search Index for DF in Zhongshan; SD: Standard Deviation.

**Table 2:** The accuracy of the classification tree models in Guangzhou and Zhongshan.

Study sites	Sensitivity	Specificity	Consistency rate
Guangzhou	87.719	92.647	91.570
Zhongshan	96.296	94.444	94.636
