# Supplementary material for: Using Baidu Search Index to Predict Dengue Outbreak in China
Source: Sci Rep. 2016 Dec 1;6:38040. doi: 10.1038/srep38040 (PMC5131307; doi:10.1038/srep38040)
Supplement: Supplementary Information [file srep38040-s1.pdf]

Supplementary Information

Using Baidu Search Index to Predict Dengue Outbreak in China

Kangkang Liu[1,2,3], Tao Wang[2,4], Zhicong Yang[5], Xiaodong Huang[3], Gabriel J Milinovich[3,6], Yi Lu[7], Qinlong Jing[1,2,5], Yao Xia[1,2], Zhengyang Zhao[5], Yang Yang[1,2,9], Shilu Tong[3], Wenbiao Hu[3*], Jiahai Lu[1,2,8*]

[1] School of Public Health, Key Laboratory of Tropical Diseases Control of Ministry of Education, Sun Yat-Sen University, Guangzhou, P. R. China.

[2] Zhongshan Research Institute of Public Health, School of Public Health, Sun Yat-Sen University, Zhongshan, P.R China.

[3] School of Public Health and Social Work, Institute of Health and Biomedical Innovation, Queensland University of Technology, Brisbane, Australia.

[4] Zhongshan Center for Diseases Control and Prevention, Zhongshan, P.R China.

[5] Guangzhou Center for Disease Control and Prevention, Guangzhou, P.R China.

[6] School of Medicine, University of Queensland, Brisbane, Australia.

[7] School of Public Health, University at Albany, State University of New York, Albany, United States.

[8] Institute of Emergency Technology for Serious Infectious Diseases Control and Prevention, Guangdong Provincial Department of Science and Technology; Emergency Management Office, the People's Government of Guangdong Province, Guangzhou, P. R. China.

[9] The School of Food Science, Guangdong Pharmaceutical University, Guangzhou, P. R. China.

[*] Correspondence and requests for materials should be addressed to J.L. (email: lujiahai@mail.sysu.edu.cn) or W.H. (email: w2.hu@qut.edu.au)

Table S1 Cross-correlations between the weekly DF incidence rates and the weekly BSI for DF in Guangzhou and Zhongshan from 1 January 2010 to 31 December 2014.

Guangzhou			Zhongshan		
Lag (weeks)	Cross Correlation	Std. Error	Lag (weeks)	Cross Correlation	Std. Error
0	0.924*	0.062	0	0.698*	0.062
1	0.904*	0.062	1	0.658*	0.062
2	0.717*	0.062	2	0.647*	0.062
3	0.505*	0.062	3	0.533*	0.062
4	0.332*	0.062	4	0.489*	0.062
5	0.227*	0.063	5	0.437*	0.063
6	0.151*	0.063	6	0.369*	0.063
7	0.093	0.063	7	0.385*	0.063
8	0.052	0.063	8	0.407*	0.063
9	0.025	0.063	9	0.417*	0.063
10	0.006	0.063	10	0.373*	0.063
11	-0.007	0.063	11	0.302*	0.063
12	-0.013	0.063	12	0.215*	0.063
13	-0.012	0.064	13	0.171*	0.064
14	-0.011	0.064	14	0.136*	0.064
15	-0.006	0.064	15	0.067	0.064
16	-0.006	0.064	16	0.030	0.064
17	-0.006	0.064	17	0.004	0.064
18	-0.006	0.064	18	-0.003	0.064
19	-0.007	0.064	19	-0.016	0.064
20	-0.007	0.064	20	-0.021	0.064

*: $P < 0.05$

Table S2 DF incidence rate percentiles in Guangzhou and Zhongshan, China from 2010 to 2014.

Percentiles (%)	Guangzhou ($1/10^5$)	Zhongshan ($1/10^5$)
25	0.000	0.000
50	0.000	0.000
75	0.031	0.000
80	0.056	0.000
85	0.121	0.063
90	0.476	0.442

Table S3 Spearman's correlations between weekly DF incidence rates and weekly Baidu search query data in Guangzhou and Zhongshan, China from 1 January, 2010 to 31 December 2014.

	DF incidence_GZ	DF incidence_ZS	BDI_GZ	BDI_ZS
ADF_GZ	1.000	0.763^{**}	0.687^{**}	0.729^{**}
ADF_ZS	0.763^{**}	1.000	0.607^{**}	0.800^{**}
BSI_GZ	0.687^{**}	0.607^{**}	1.000	0.064^{**}
BSI_ZS	0.729^{**}	0.800^{**}	0.604^{**}	1.000

**: $P < 0.01$

ADF_GZ: Autochthonous DF in Guangzhou;

ADF_ZS: Autochthonous DF in Zhongshan;

BSI_GZ: Baidu DF search index in Guangzhou;

BSI_ZS: Baidu DF search index in Zhongshan.

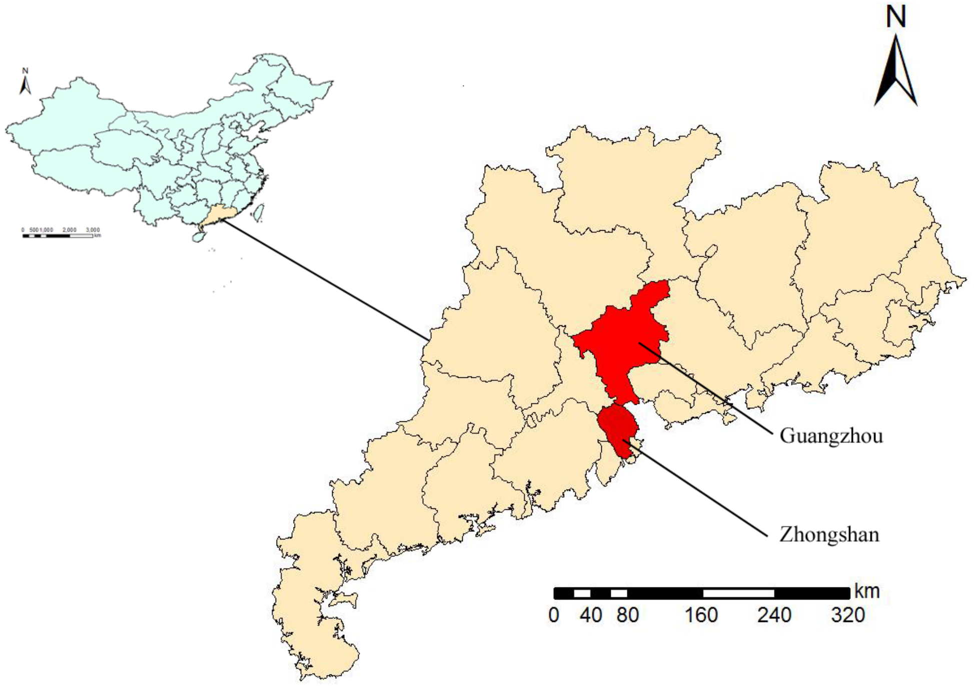

Figure S1 Locations of Guangzhou and Zhongshan, Guangdong province, China.
(It was created with ArcGIS software version 10.3.1, http://www.esri.com/software/arcgis.)

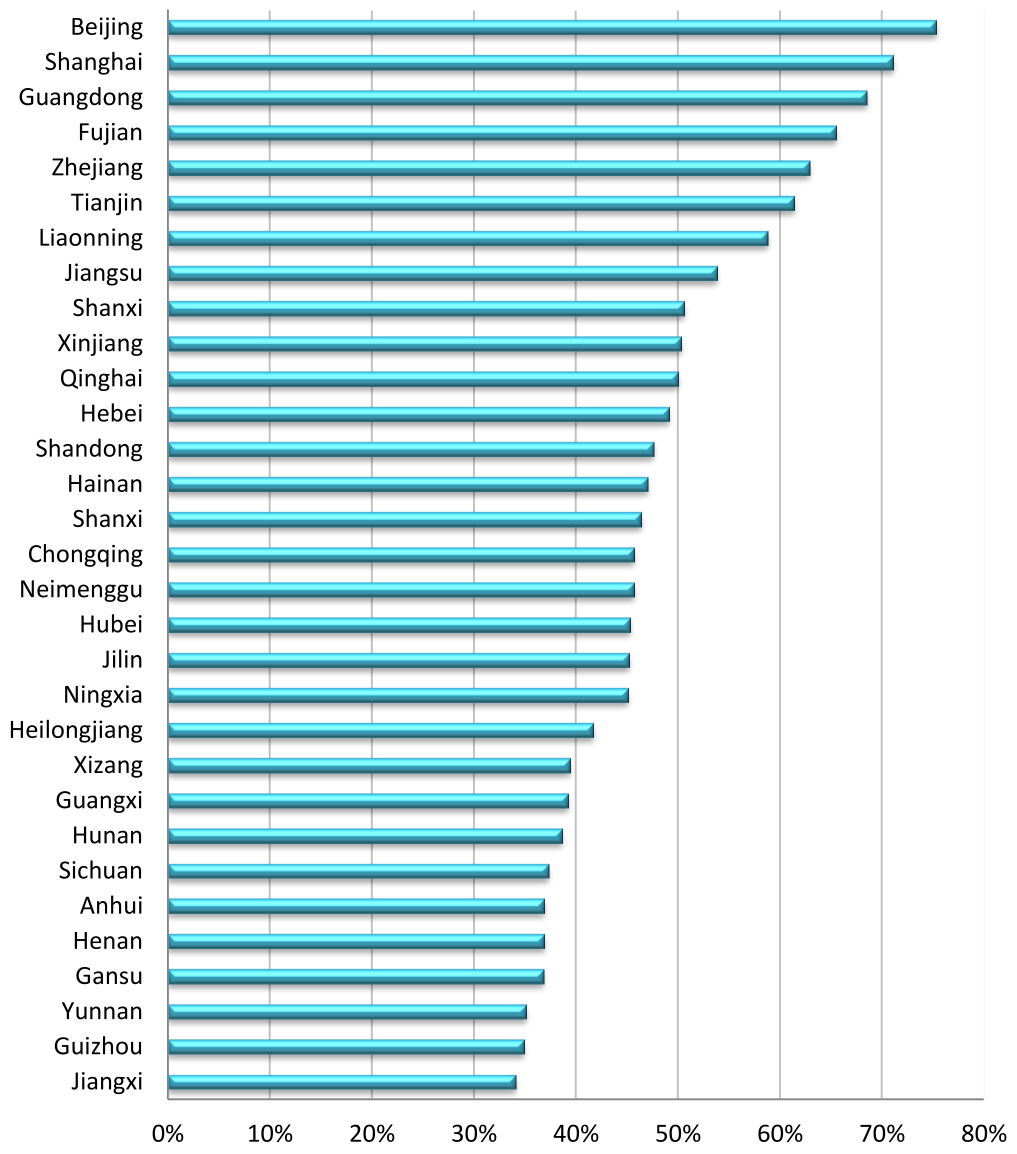

Figure S2 Internet penetration rates in different province of China in 2014.
